# Supplementary material for: Resolving the full spectrum of human genome variation using Linked-Reads
Source: Genome Res. 2019 Apr;29(4):635–45. doi: 10.1101/gr.234443.118 (PMC6442396; doi:10.1101/gr.234443.118)
Supplement: Supplemental Material [file supp_gr.234443.118_Supplemental_longranger-2.2.zip › longranger-2.2.tar.bz2!/lr2.2-public/lib/stan/cmdstan/stan/doc/api/html/agrad_2rev_2matrix_2log__determinant__spd_8hpp_source.html]

Stan: src/stan/agrad/rev/matrix/log\_determinant\_spd.hpp Source File


|  |  |
| --- | --- |
|  | Stan  2.5.0  probability, sampling & optimization |


- Main Page
- Related Pages
- Namespaces
- Classes
- Files

- File List
- File Members

All Classes Namespaces Files Functions Variables Typedefs Enumerations Enumerator Friends Macros Pages

- src
- stan
- agrad
- rev
- matrix

log\_determinant\_spd.hpp

Go to the documentation of this file.

1 #ifndef STAN\_\_AGRAD\_\_REV\_\_MATRIX\_\_LOG\_DETERMINANT\_SPD\_HPP

2 #define STAN\_\_AGRAD\_\_REV\_\_MATRIX\_\_LOG\_DETERMINANT\_SPD\_HPP

3

4 #include <vector>

5 #include <stan/math/error\_handling/dom\_err.hpp>

6 #include <stan/math/matrix/Eigen.hpp>

7 #include <stan/math/matrix/typedefs.hpp>

8 #include <stan/agrad/rev/var.hpp>

9 #include <stan/agrad/rev/matrix/typedefs.hpp>

10 #include <stan/math/error\_handling/matrix/check\_square.hpp>

11

12 // FIXME: use explicit files

13 #include <stan/agrad/rev.hpp>

14

15 namespace stan {

16  namespace agrad {

17

18  namespace {

19  template <int R,int C>

20  class log\_determinant\_spd\_alloc : public chainable\_alloc {

21  public:

22  virtual ~log\_determinant\_spd\_alloc() {}

23

24  Eigen::Matrix<double,R,C> \_invA;

25  };

26

27

28  template<int R,int C>

29  class log\_determinant\_spd\_vari : public vari {

30  log\_determinant\_spd\_alloc<R,C> \*\_alloc;

31  int \_rows;

32  int \_cols;

33  vari\*\* \_adjARef;

34  public:

35  log\_determinant\_spd\_vari(const Eigen::Matrix<var,R,C> &A)

36  : vari(log\_determinant\_spd\_vari\_calc(A,&\_alloc)),

37  \_rows(A.rows()),

38  \_cols(A.cols()),

39  \_adjARef((vari\*\*)stan::agrad::memalloc\_.alloc(sizeof(vari\*)

40  \* A.rows() \* A.cols()))

41  {

42  size\_t pos = 0;

43  for (size\_type j = 0; j < \_cols; j++) {

44  for (size\_type i = 0; i < \_rows; i++) {

45  \_adjARef[pos++] = A(i,j).vi\_;

46  }

47  }

48  }

49

50  static

51  double log\_determinant\_spd\_vari\_calc(const Eigen::Matrix<var,R,C> &A,

52  log\_determinant\_spd\_alloc<R,C> \*\*alloc)

53  {

54  using stan::math::dom\_err;

55

56  // allocate space for information needed in chain

57  \*alloc = new log\_determinant\_spd\_alloc<R,C>();

58

59  // compute cholesky decomposition of A

60  (\*alloc)->\_invA.resize(A.rows(),A.cols());

61  for (size\_type j = 0; j < A.cols(); j++)

62  for (size\_type i = 0; i < A.rows(); i++)

63  (\*alloc)->\_invA(i,j) = A(i,j).val();

64  Eigen::LDLT< Eigen::Matrix<double,R,C> > ldlt((\*alloc)->\_invA);

65  if (ldlt.info() != Eigen::Success) {

66  // Handle this better.

67  (\*alloc)->\_invA.setZero(A.rows(),A.cols());

68  double y = 0;

69  double result = -std::numeric\_limits<double>::infinity();

70  return dom\_err("log\_determinant\_spd(%1%)",

71  y,

72  "matrix argument",

73  "failed LDLT factorization","",

74  &result);

75  }

76

77  // compute the inverse of A (needed for the derivative)

78  (\*alloc)->\_invA.setIdentity(A.rows(),A.cols());

79  ldlt.solveInPlace((\*alloc)->\_invA);

80

81  if (ldlt.isNegative() || (ldlt.vectorD().array() <= 1e-16).any()) {

82  double y = 0;

83  double result = -std::numeric\_limits<double>::infinity();

84  return dom\_err("log\_determinant\_spd(%1%)",

85  y,

86  "matrix argument",

87  "matrix is negative definite","",

88  &result);

89  }

90

91  double ret = ldlt.vectorD().array().log().sum();

92  if (!boost::math::isfinite(ret)) {

93  double y = 0;

94  double result = -std::numeric\_limits<double>::infinity();

95  return dom\_err("log\_determinant\_spd(%1%)",

96  y,

97  "matrix argument",

98  "log determininant is infinite","",

99  &result);

100  }

101  return ret;

102  }

103

104  virtual void chain() {

105  size\_t pos = 0;

106  for (size\_type j = 0; j < \_cols; j++) {

107  for (size\_type i = 0; i < \_rows; i++) {

108  \_adjARef[pos++]->adj\_ += adj\_\*\_alloc->\_invA(i,j);

109  }

110  }

111  }

112  };

113  }

114

115  template <int R, int C>

116  inline var log\_determinant\_spd(const Eigen::Matrix<var,R,C>& m) {

117  stan::math::check\_square("log\_determinant\_spd(%1%)",m,"m",(double\*)0);

118  return var(new log\_determinant\_spd\_vari<R,C>(m));

119  }

120

121  }

122 }

123 #endif

\_alloc

log\_determinant\_spd\_alloc< R, C > \* \_alloc

**Definition:** log\_determinant\_spd.hpp:30

dom\_err.hpp

stan::math::dom\_err

bool dom\_err(const char \*function, const T &y, const char \*name, const char \*error\_msg, const T\_msg error\_msg2, T\_result \*result)

**Definition:** dom\_err.hpp:36

typedefs.hpp

typedefs.hpp

boost::math::isfinite

bool isfinite(const stan::agrad::var &v)

Checks if the given number has finite value.

**Definition:** boost\_fpclassify.hpp:39

stan::agrad::memalloc\_

memory::stack\_alloc memalloc\_

**Definition:** var\_stack.cpp:16

stan::agrad::log\_determinant\_spd

var log\_determinant\_spd(const Eigen::Matrix< var, R, C > &m)

**Definition:** log\_determinant\_spd.hpp:116

\_cols

int \_cols

**Definition:** log\_determinant\_spd.hpp:32

\_adjARef

vari \*\* \_adjARef

**Definition:** log\_determinant\_spd.hpp:33

rev.hpp

stan::agrad::size\_type

Eigen::Matrix< double, Eigen::Dynamic, Eigen::Dynamic >::Index size\_type

**Definition:** typedefs.hpp:14

stan::math::rows

size\_t rows(const Eigen::Matrix< T, R, C > &m)

**Definition:** rows.hpp:12

var.hpp

stan::math::cols

size\_t cols(const Eigen::Matrix< T, R, C > &m)

**Definition:** cols.hpp:12

\_invA

Eigen::Matrix< double, R, C > \_invA

**Definition:** log\_determinant\_spd.hpp:24

\_rows

int \_rows

**Definition:** log\_determinant\_spd.hpp:31

Eigen.hpp

stan::agrad::var

Independent (input) and dependent (output) variables for gradients.

**Definition:** var.hpp:27

stan::math::e

double e()

Return the base of the natural logarithm.

**Definition:** constants.hpp:86

stan::math::check\_square

bool check\_square(const char \*function, const Eigen::Matrix< T\_y, Eigen::Dynamic, Eigen::Dynamic > &y, const char \*name, T\_result \*result)

Return true if the specified matrix is square.

**Definition:** check\_square.hpp:24

check\_square.hpp


---

[ Stan Home Page ]

*© 2011–2014,
Stan Development Team.*
